# Historical baleen plates indicate that once abundant Antarctic blue and fin whales demonstrated distinct migratory and foraging strategies

**DOI:** 10.1002/ece3.11376

**Published:** 2024-05-06

**Authors:** Malia E. K. Smith, John J. Ososky, Kathleen E. Hunt, William R. Cioffi, Andy J. Read, Ari S. Friedlaender, Matt McCarthy, Alyson H. Fleming

**Affiliations:** ^1^ Department of Biology and Marine Biology University of North Carolina Wilmington Wilmington North Carolina USA; ^2^ National Museum of Natural History Smithsonian Institution Washington District of Columbia USA; ^3^ Smithsonian‐Mason School of Conservation & George Mason University Front Royal Virginia USA; ^4^ Nicholas School of the Environment Duke University Marine Laboratory Beaufort North Carolina USA; ^5^ Institute of Marine Sciences University of California Santa Cruz Santa Cruz California USA; ^6^ Forest and Wildlife Ecology University of Wisconsin Madison Madison Wisconsin USA

**Keywords:** Antarctic blue whale, Antarctic fin whale, feeding ecology, migration, niche partitioning, Southern Ocean, stable isotopes

## Abstract

Southern hemisphere blue (*Balaenoptera musculus intermedia*) and fin (*Balaenoptera physalus*) whales are the largest predators in the Southern Ocean, with similarities in morphology and distribution. Yet, understanding of their life history and foraging is limited due to current low abundances and limited ecological data. To address these gaps, historic Antarctic blue (*n* = 5) and fin (*n* = 5) whale baleen plates, collected in 1947–1948 and recently rediscovered in the Smithsonian National Museum of Natural History, were analyzed for bulk (δ^13^C and δ^15^N) stable isotopes. Regular oscillations in isotopic ratios, interpreted as annual cycles, revealed that baleen plates contain approximately 6 years (14.35 ± 1.20 cm year^−1^) of life history data in blue whales and 4 years (16.52 ± 1.86 cm year^−1^) in fin whales. Isotopic results suggest that: (1) while in the Southern Ocean, blue and fin whales likely fed at the same trophic level but demonstrated niche differentiation; (2) fin whales appear to have had more regular annual migrations; and (3) fin whales may have migrated to ecologically distinct sub‐Antarctic waters annually while some blue whales may have resided year‐round in the Southern Ocean. These results reveal differences in ecological niche and life history strategies between Antarctic blue and fin whales during a time period when their populations were more abundant than today, and before major human‐driven climatic changes occurred in the Southern Ocean.

## Graphical abstract

Our study details the ecological differences between historic Antarctic blue and fin whales using stable isotope analysis on baleen powder. We provide evidence in support of two separate migratory and foraging patterns (one for each species) using a different technique than previous studies, which used observations, acoustics, or genetics. This is the first study to analyze baleen from these Antarctic species.

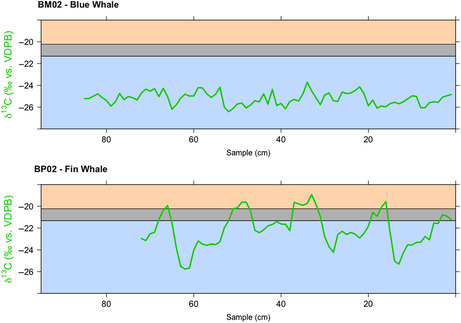

## 1 INTRODUCTION

The pre‐exploitation abundance of Antarctic blue whales (*Balaenoptera musculus intermedia*) and fin whales (*Balaenoptera physalus*) has been estimated at ~200,000–300,000 (Branch et al., 2004) and ~400,000–600,000 (Gambell, 1976), respectively. Both populations experienced dramatic declines due to pelagic commercial whaling in the Southern Ocean (Clapham & Baker, 2002). These declines accelerated after World War I, due to a variety of technological advancements (Gambell, 1976) that allowed whalers to target the largest cetacean species, such as blue and fin whales, which were the most profitable to harvest. Exploitation of blue and fin whales in the Southern Ocean ended in the mid‐1960s and 1976, respectively, through legal protection under the International Convention for the Regulation of Whaling (Best, 1993; IWC, 1978). By the end of the 20th century, it is estimated that over one million Antarctic blue and fin whales had been killed (Rocha et al., 2014). Today, the Antarctic blue whale stock is estimated at ~3000 and listed as critically endangered (Cooke, 2018), and the fin whale stock is estimated to be ~15,000–40,000 and listed as vulnerable on the IUCN Red List (Cooke, 2018). The low abundance of both species has made modern scientific studies difficult and thus, much of what is known about these two species comes from whaling‐era data and reports (Branch, Stafford, et al., 2007; Nemoto, 1959; Nishiwaki & Hayashi, 1950). In particular, the winter distribution, year‐round foraging ecology, and migration behavior of these species remain poorly described. As the largest predators in the Southern Ocean, and major consumers of ecologically important Antarctic krill (Kawamura, 1980; Savoca et al., 2021), understanding their foraging behavior and movement patterns is important to understanding and managing this ecosystem.

Antarctic blue and fin whales are known to feed predominantly on the krill species *Euphausia superba* and, to a lesser extent, on *Thysanoessa macrura* particularly in some regions of the Southern Ocean (Kawamura, 1980; Nemoto, 1962; Nemoto & Nasu, 1958). Some differences in krill size or foraging location have been suggested based on whaling data (Laws, 1977; Nemoto, 1959) yet further details on niche partitioning between these species remain unknown.

Most contemporary data on Antarctic blue and fin whale distribution comes from studies employing passive acoustics and line‐transect surveys (Shabangu et al., 2020), the combination of which provides a more complete year‐round picture of a species distribution (Branch, Stafford, et al., 2007; Fleming et al., 2018; Širović et al., 2009). Acoustic recordings have detected blue whale calls in the Southern Ocean year‐round (Double et al., 2013; Širović et al., 2009). In the southwest Pacific sector, primarily around Australia and New Zealand, Antarctic blue whales have been recorded, with greater presence in fall, winter, and spring months (Balcazar et al., 2017; Stafford et al., 2004). Seasonal presence has also been detected in the austral fall and winter in the central and eastern Indian Ocean (Stafford et al., 2004; Truong & Rogers, 2021), the west coast of Africa (Best, 1998; Figueiredo & Weir, 2014), and the eastern tropical Pacific (Stafford et al., 2004; Truong & Rogers, 2021). Collectively, evidence to date suggests that Antarctic blue whales primarily feed in the Southern Ocean in the summer months with some portion of the population migrating to lower latitudes in the winter months. However, some individuals may not migrate (Branch, 2007; Risting, 1928; Širović et al., 2009; Thomisch et al., 2016) and, for numerous blue whale populations, feeding may occur at multiple locations (Double et al., 2013).

For fin whales, acoustic, observational, and catch data suggest that individuals likely occur in the Southern Ocean only seasonally (Chapman, 1974; Miyashita et al., 1995). Calling activity is highest between February and July (Aulich et al., 2019; Širović et al., 2009) with geographically variable presence around the Southern Ocean. At lower latitudes, calls have been recorded in the austral fall, winter, and spring. Populations of fin whales have been detected along both coasts of Australia from April–October, with higher call densities in biologically productive areas like the Perth Canyon where feeding may occur (Aulich et al., 2019). A recent acoustic study suggested that some fin whales could be using New Zealand as a wintering or breeding ground (Constaratas et al., 2021).

In addition to gaps in our understanding of modern populations of Antarctic blue and fin whales, it is also unclear whether feeding patterns observed today might differ from the species' recent evolutionary past and may be influenced by changes in climate and ecosystem conditions. While the current low population sizes are the result of decades of intense over‐harvest, new insights on these species may also be feasible because of this whaling legacy. Specifically, a collection of Antarctic blue and fin whale baleen plates obtained during whaling expeditions was recently rediscovered in the Smithsonian's National Museum of Natural History (NMNH; Potter et al., 2016). The collection includes baleen plates from more than 1600 Antarctic blue and fin whales. These plates were collected from the 1946–1947 and 1947–1948 Supreme Commander of the Allied Powers (SCAP) whaling expeditions in the Southern Ocean (Hall, 2019; Nishiwaki & Hayashi, 1950; Potter et al., 2016); shipboard observers were instructed to collect the two longest baleen plates from each whale. This collection also includes detailed metadata from the whaling logs (see Appendix A).

Stable isotope analysis, commonly used in ecological studies to infer diet, trophic level, and foraging location (Hobson et al., 1994; West et al., 2006), can be applied to archived baleen samples to provide insight into an individual whale's movement and ecology prior to death (Busquets‐Vass et al., 2017; Schell et al., 1989a, 1989b). Baleen plates grow continuously from a proximal base embedded in gum tissue of the upper palate, eroding away simultaneously at the distal tip, recording information about an animal's life history, with timelines ranging from approximately 2 (gray whales, *Eschrichtius robustus*; Caraveo‐Patiño et al., 2007) to 20 years (bowhead whales, *Balaena mysticetus*; Schell et al., 1989a), depending on species‐specific baleen length and growth rates. These timelines may be verified with stable isotope data because stable isotope ratios often display oscillations along the length of the baleen plate, representing annual migrations (Busquets‐Vass et al., 2017; Matthews & Ferguson, 2015; Ryan et al., 2014; Schell et al., 1989a, 1989b). Thus, a single plate of baleen provides a multi‐year time series for each individual animal, analogous to repeated sampling, potentially containing valuable clues as to the animal's prey selection and movement patterns across multiple years.

Using the archived baleen tissue from the SCAP whaling expeditions, we investigated the life histories of ten individual blue and fin whales harvested 70 years ago. At that time, significant population declines had already occurred, but both species were significantly more abundant than they are today, and the most significant impacts of anthropogenic climate change were not yet apparent. To our knowledge, these are the first baleen plates from blue whales from the Southern Ocean to be analyzed (see Buss et al., 2022 for fin whale baleen isotopes collected at South Georgia). The main objectives of this study are to (1) examine the foraging niche of both Antarctic blue and fin whales of the 1940s by assessing isotopic differences between and within species and (2) compare the migratory behavior and habitat use of Antarctic blue and fin whales of the 1940s.

## 2 MATERIALS AND METHODS

### 2.1 Stable isotope analysis

**FIGURE 1:**
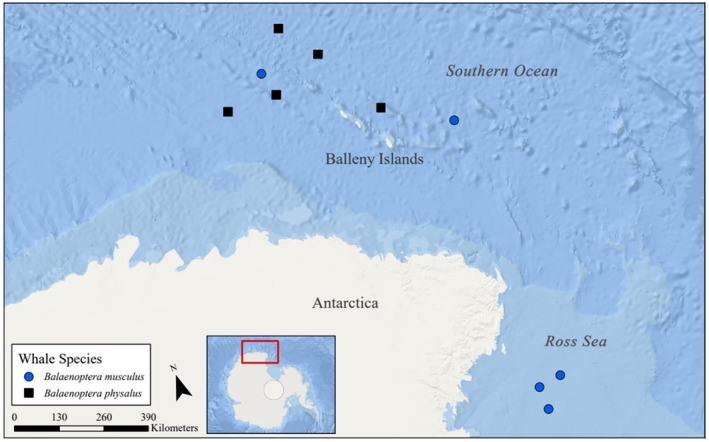
Map of Antarctica showing the locations where individual Antarctic blue (*Balaenoptera musculus intermedia*; *n* = 5; blue circles) and fin whales (*Balaenoptera physalus*; *n* = 5; black square) were harvested.

Five baleen plates from each species (ten in total) were selected from the NMNH collection with similar demographic and geographic representation (see Appendix; Figure 1). Each plate was sampled at 1 cm intervals penetrating only the cortex layer (as recommended by Rita et al., 2019) along the buccal edge using a Dremel tool to collect ~3.0 mg of powder. The powder was analyzed for bulk δ^13^C and δ^15^N values at the Isotope Ratio Mass Spectrometry Core Facility in the Center for Marine Science at the University of North Carolina Wilmington (see Appendix for details). The instrument error for bulk δ^13^C and δ^15^N values from the USGS 40 standard runs (*n* = 201) was 0.32‰ and 0.21‰, respectively, and for USGS 41 (*n* = 249) was 0.37‰ and 0. 27‰, respectively. We calculated the mass and atomic C:N ratio using the formulas (%C/%N) and $\left(\frac{14}{12}\times \frac{\%C}{\%N}\right)$, respectively. Samples were not corrected for the Suess Effect (Suess, 1955) due to the minimal changes of δ^13^C values (−0.005‰ ± 0.003‰ year^−1^) in the Southern Ocean (Eide et al., 2017; McNeil et al., 2001).

### 2.2 Statistical analyses

#### 2.2.1 Baleen growth rates

To estimate species‐specific baleen growth rates, both δ^13^C and δ^15^N values were analyzed for presence of putative annual cycles. Carbon data demonstrated regular cycles in only two whales, while nitrogen data demonstrated better‐resolved cycles in most individuals, especially in the blue whales. Furthermore, several recent studies have shown relatively consistent δ^15^N values of annual cycles within baleen plates of blue whales and other mysticetes (Blevins et al., 2022; Busquets‐Vass et al., 2017; Eisenmann et al., 2016; Silva et al., 2019; Trueman et al., 2019). We detrended each plate's δ^15^N values using a Gaussian low‐pass filter to study seasonal trends (Park & Gambéroni, 1995; Pomerleau et al., 2018) then used Fast Fourier Transform (FFT) to calculate peak spectral frequencies to estimate annual cycles (Figure A5). Spectral peak frequencies were converted to cm/cycle (1/frequency) for each individual, then averaged by species to calculate species growth rate (Matthews & Ferguson, 2015; Pomerleau et al., 2018). Only individuals with at least three annual cycles were considered when calculating species averages (shown as ±1 standard deviation).

#### 2.2.2 Niche comparison between blue and fin whales

Normality and homogeneity of variance were first examined with Shapiro–Wilk's and Levene's test, respectively. While fin whales showed equal variance across both isotopes, blue whales did not. Much of the data followed a normal distribution but not all individual plates did and thus a Mann–Whitney *U* test was used to examine statistical differences in both isotopes at the species level. All statistical tests mentioned above were performed using the stats (R Core Team, 2020) and car (Fox & Weisberg, 2019) packages in R version 4.0.3.

To examine and compare the ecological niche space of blue and fin whales, standard area bivariate ellipses were estimated through Bayesian methods using the Stable Isotope Bayesian Ellipses in R (SIBER) (Jackson et al., 2011; Jackson & Parnell, 2023). SIBER uses Markov‐Chain Monte Carlo (MCMC) simulations to construct the parameters of ellipses, incorporating estimates of uncertainty through numerous iterations. Here, maximum likelihood estimates corrected for small sample size (SEA_C_) and Bayesian estimates of isotopic niche size (SEA_B_) were used to compare species niche space and overlap in two different ways. First, all values from blues and fins were compared, grouped by species. Second, values were further grouped by identifying isotopic peaks and valleys, thereby comparing both species and their corresponding seasonal habitat isotopic values. Data for each peak and valley were chosen based on the highest and lowest adjacent 2–3 values from each carbon oscillation. Niche size and overlap were measured twice for both SEA_C_ and SEA_B_ first incorporating 40% of samples and second incorporating 95%, reflecting the core trophic niche and full trophic niche, respectively (Buss et al., 2022; Jones et al., 2020). For the SEA_B_ calculations, MCMC parameters were 20,000 iterations, 1000 burn‐in, 10% thinning, and two independent chains. Species and seasonal habitat differences were evaluated by examining overlap of the 95% credible intervals of the posterior distributions (95% CIs). Differences by sex could not be tested due to small sample size. All plots were generated within SIBER or other stats and graphics packages in R version 4.0.3 (R Core Team, 2020).

Importantly, this study used many repeated measurements from each individual, which violates the assumption of independence of individual data points. To address this, additional datasets were created by randomly subsampling each baleen plate 40 times, creating 5 new datasets of *n* = 400 samples (Buss et al., 2022). Autocorrelation was then examined within each dataset using the Breusch‐Godfrey test (Breusch, 1978; Zeileis & Hothorn, 2002), and no significant correlation was found. The 95% CIs of posterior distributions from the SEA_B_s were then compared to each other and the original full dataset to assess the influence of pseudoreplication. The results were similar and thus the original dataset is presented below.

#### 2.2.3 Habitat use

**FIGURE A1:**
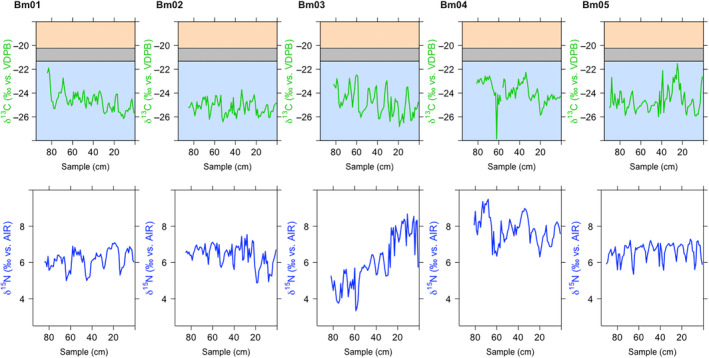
The stable carbon (δ^13^C, green, top row) and nitrogen (δ^15^N, blue, bottom row) isotope values along the length of five Antarctic blue whales (*Balaenoptera musculus intermedia*) baleen plates from individuals BM01‐BM05. Stable isotope values are on the y‐axis, and the x‐axis shows the sampling progression along the plates from old to new growth (left to right: distal end of plate to proximal end of plate). In the top row, the Subantarctic Front (Espinasse et al., 2019) is represented by the shaded orange area, seasonal variation is in gray shading, and the Southern Ocean is in light blue. Shading is corrected based on the diet‐tissue discrimination factors of POM to skin (Seyboth et al., 2018) to baleen (Borrell et al., 2012) for fin whales (Δ^13^C = 2.68‰).

**FIGURE A2:**
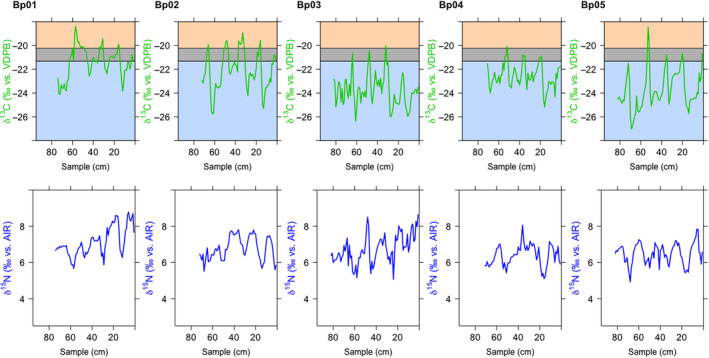
The stable carbon (δ^13^C, green, top row) and nitrogen (δ^15^N, blue, bottom row) isotope values along the length of five fin whale (*Balaenoptera physalus*) baleen plates from individuals BP01‐BP05. Stable isotope values are on the y‐axis, and the x‐axis shows the sampling progression along the plates from old to new growth (left to right: distal end of plate to proximal end of plate). In the top row, the Subantarctic Front (Espinasse et al., 2019) is represented by the shaded orange area, seasonal variation is in gray shading, and the Southern Ocean is in light blue. Shading is corrected based on the diet‐tissue discrimination factors of POM to skin (Seyboth et al., 2018) to baleen (Borrell et al., 2012) for fin whales (Δ^13^C = 2.68‰).

To assess the habitat use patterns of both species, baleen isotope values were compared to published particulate organic matter (POM) isoscape data for the region where the specimens were first captured (Espinasse et al., 2019). Before comparing, blue and fin whale δ^13^C values were first corrected based on the diet‐tissue discrimination factor of POM to skin (Δ^13^C = 1.7‰, Seyboth et al., 2018) and skin to baleen (Δ^13^C = 0.98, Borrell et al., 2012) for fin whales, which combine to an overall Δ^13^C value of 2.68‰ (Figure 3, Figures A1, A2). Based on the isoscapes from Espinasse et al. (2019), baleen carbon peaks were interpreted as residence in, or migration towards, potential breeding grounds near the Subantarctic Front, while the carbon valleys were interpreted as residence or migration within the Southern Ocean, or potential feeding grounds (Figure 3). To examine the contribution of potential prey items to blue and fin isotopic signatures, isotope values of likely prey species were collected from the literature (Eisenmann et al., 2016; Quillfeldt et al., 2008; Schmidt et al., 2003; Torres et al., 2015). The krill to baleen diet‐tissue discrimination factor from Borrell et al. (2012) was then used to visualize the baleen δ^13^C and δ^15^N values (Δ^15^N = 2.77 ± 0.22; Borrell et al., 2012) in relation to these prey (Figure 3).

Finally, cross‐correlation functions (CCF) were used to assess correlations and potential synchronous relationships between detrended δ^13^C and δ^15^N values for each plate to examine if changes in isotopes were temporally and, thus potentially geographically, synchronous.

## 3 RESULTS

### 3.1 Baleen growth rates

Blue whales had longer baleen plates ranging from 81 to 90 cm, while fin whale plates ranged from 59–83 cm. Based on our growth rate calculations, the typical plate from a blue whale baleen in our study contained approximately 6 years of life history (14.35 ± 1.20 cm year^−1^; Table 1), similar to previous estimates from northern hemisphere populations (Busquets‐Vass et al., 2017). Fin whale baleen contained approximately 4 years of growth (16.52 ± 1.86 cm year^−1^; Table 1), with an annual baleen growth rate slower than that calculated by Bentaleb et al. (2011); (20.1 cm year^−1^) from northern hemisphere fin whales, which are smaller (Brodie, 1975). Only 2 blue whales (one male, one female) and 3 fin whales (one male, one non‐pregnant female, and one female pregnant at death) had more than 2 detectable isotopic cycles after detrending and FFT methods were applied (Table 1). The mass and atomic C:N ratios for blue (3.4 ± 0.1 and 3.9 ± 0.1, respectively; Table 1) and fin whales (3.4 ± 0.0 and 3.9 ± 0.0, respectively; Table 1) were similar to values reported in previous studies on blue and fin whales from the northern hemisphere (Busquets‐Vass et al., 2017; Ryan et al., 2014).

**TABLE 1:** Antarctic blue (*Balaenoptera musculus intermedia*) and fin (*Balaenoptera physalus*) whale baleen data.

Sample ID	USNM specimen number	Species	Date captured	Latitude	Longitude	Sex	Pregnant	# of subsamples	Mass C:N ratio	Atomic C:N ratio	Number of nitrogen cycles	Baleen growth rate (cm year^−1^)
Bm01	617,418	*Balaenoptera musculus intermedia*	1/4/1948	−64.67	158.4	M	—	84	3.4 ± 0.1	3.9 ± 0.1	5	15.20
Bm02	617,632	*Balaenoptera musculus intermedia*	2/12/1948	−74.65	172.47	F	No	85	3.4 ± 0.0	3.9 ± 0.1	2	—
Bm03	617,698	*Balaenoptera musculus intermedia*	2/7/1948	−67.3	169.2	F	Yes	82	3.4 ± 0.0	3.9 ± 0.1	1	—
Bm04	617,622	*Balaenoptera musculus intermedia*	2/24/1948	−74.4	174.58	M	—	80	3.3 ± 0.1	3.9 ± 0.1	2	—
Bm05	617,640	*Balaenoptera musculus intermedia*	2/14/1948	−75.25	173.08	F	Yes	90	3.4 ± 0.0	4.0 ± 0.0	6	13.50
Bp01	617,395	*Balaenoptera physalus*	12/31/1947	−63.68	160.27	M	—	74	3.3 ± 0.0	3.9 ± 0.0	1	—
Bp02	617,471	*Balaenoptera physalus*	1/15/1948	−65.33	158.77	M	—	72	3.4 ± 0.0	3.9 ± 0.0	4	16.25
Bp03	617,458	*Balaenoptera physalus*	1/12/1948	−65.25	155.67	F	Yes	82	3.4 ± 0.1	4.0 ± 0.1	5	14.80
Bp04	617,555	*Balaenoptera physalus*	1/29/1948	−64.68	161.95	F	Yes	71	3.4 ± 0.0	3.9 ± 0.0	2	—
Bp05	617,571	*Balaenoptera physalus*	2/3/1948	−66.53	164.73	F	No	82	3.4 ± 0.0	3.9 ± 0.0	4	18.50
Species Averages		*Balaenoptera musculus intermedia*	—	—	—	—	—	—	3.4 ± 0.1	3.9 ± 0.1	5.5	14.35 ± 1.20
		*Balaenoptera physalus*	—	—	—	—	—	—	3.4 ± 0.0	3.9 ± 0.0	4.3	16.52 ± 1.86

*Note*: Metadata for each individual (date and location of capture, sex, reproductive status) were derived from ship's logs from expeditions in the Southern Ocean. Laboratory data include the number of subsamples (collected at 1 cm increments), mass and atomic C:N ratios (mean ± 1 standard deviation), the number of nitrogen cycles (peak to peak), and the calculated baleen growth rate (cm year^−1^) for each individual (if more than two cycles) and species average with ±1 standard deviation.

### 3.2 Niche comparison between blue and fin whales

Our results showed a significant statistical difference between species for both δ^13^C (W = 964.5, *p*‐value < .001) and δ^15^N values (W = 12,646, *p*‐value = .011). While both statistically different, δ^13^C values showed much greater disparity between the two species (blues −24.62‰ ± 0.97‰ and fins −22.77‰ ± 1.69‰) compared to δ^15^N values (blues 6.60‰ ± 1.02‰ and fins 6.71‰ ± 0.74‰) (Figure 4). When examining only the peaks and valleys, presumed to represent the two ends of the whales' migratory paths, we found that blue and fin whales had significantly different carbon values for peaks (blues −23.74‰ ± 0.91‰ and fins −20.85‰ ± 1.14‰, *p* < .001) and valleys (−25.57‰ ± 0.64‰ and −24.43‰ ± 1.31‰, *p* = .010). However, there were only significant differences in nitrogen values for peaks (7.05‰ ± 0.94‰ and 7.37‰ ± 0.63‰, *p* = .003), not valleys (6.00‰ ± 0.84‰ and 5.93‰ ± 0.42‰, *p* = .721) (Figure 4).

The trophic niche of fin whales was larger than blue whales (‰^2^ = 3.7 vs. 3.0), as assessed by standard ellipse area, measured by both SEA_C_ and SEA_B._ The isotopic niche overlap between blue and fin whales when assessed at the core niche level (40% of data) was 9% and when estimated with 95% of the data, there was 42% overlap (Figure 5). The niche overlap of blue and fin whale δ^13^C and δ^15^N peak values was smaller (about 17% overlap) than the area of overlap between valleys (about 33% overlap; Figure 5c, d) when assessed with 95% of the data. The core peak ellipses (40% of data) did not overlap between blue and fin whales. The ellipses for fin whale valleys (both the 40% and 95% contours) overlap with all blue whale ellipses for both peaks at valleys (across both the 40% and 95% contours).

### 3.3 Habitat use

**FIGURE 2:**
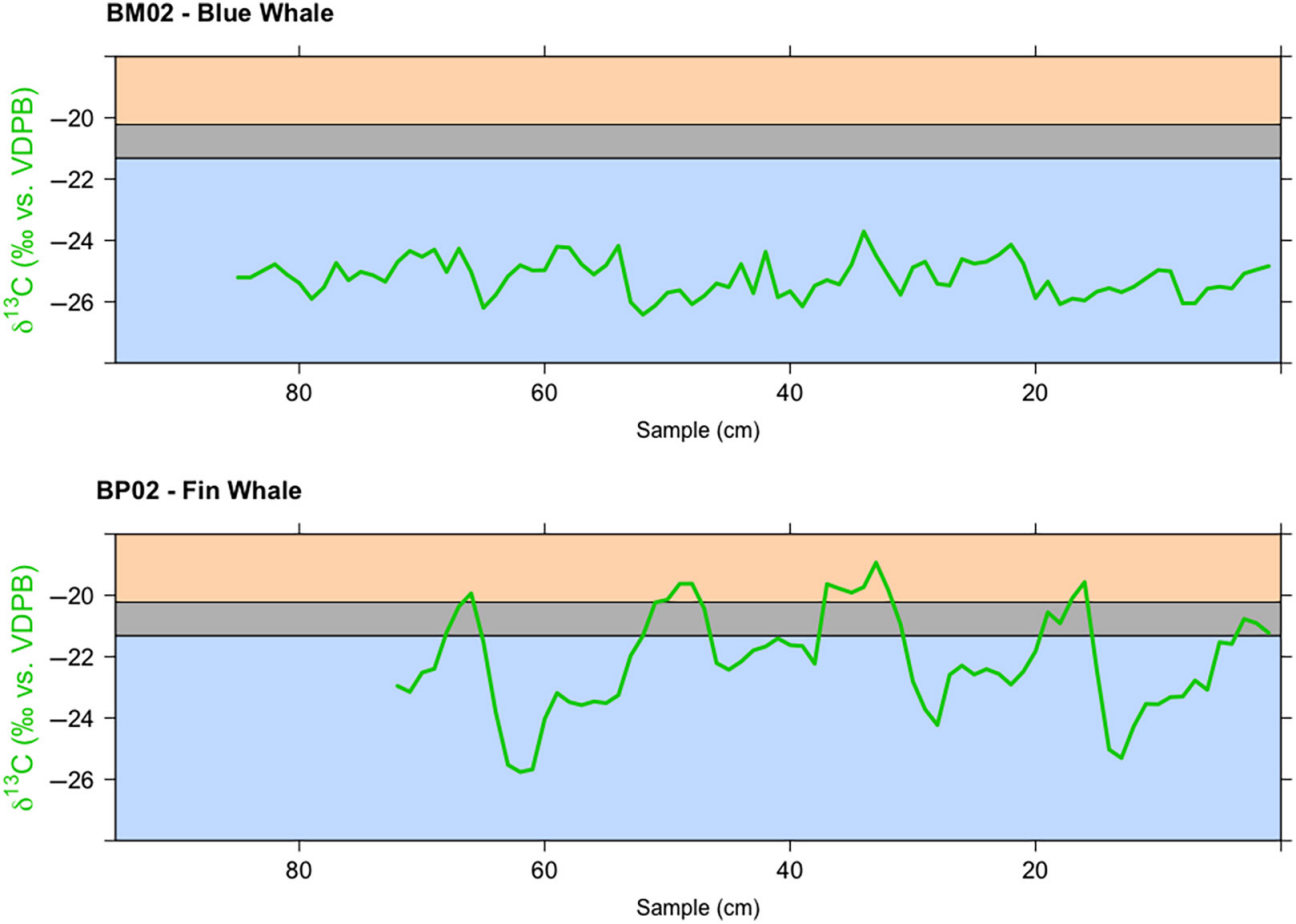
Plot of δ^13^C values (green lines) from one Antarctic blue (*Balaenoptera musculus intermedia*; individual BM02) and one Antarctic fin whale (*Balaenoptera physalus*; individual BP02) baleen plate. The x‐axis shows the sample location along the baleen plate, where 0 cm represents the proximal base of the plate (newest baleen); time goes left to right along the x‐axis, from old growth (left) to new growth (right). Isotopic values consistent with foraging in the Subantarctic Front are indicated with orange shading, and those consistent with Southern Ocean locations with light blue shading, with gray shading indicating intermediate values; shading thresholds are based on particulate organic matter δ^13^C values from Espinasse et al., 2019, with corrections based on diet‐tissue discrimination factors of POM to skin (Seyboth et al., 2018) to baleen (Borrell et al., 2012) for fin whales (Δ^13^C = 2.68‰).

**FIGURE 3:**
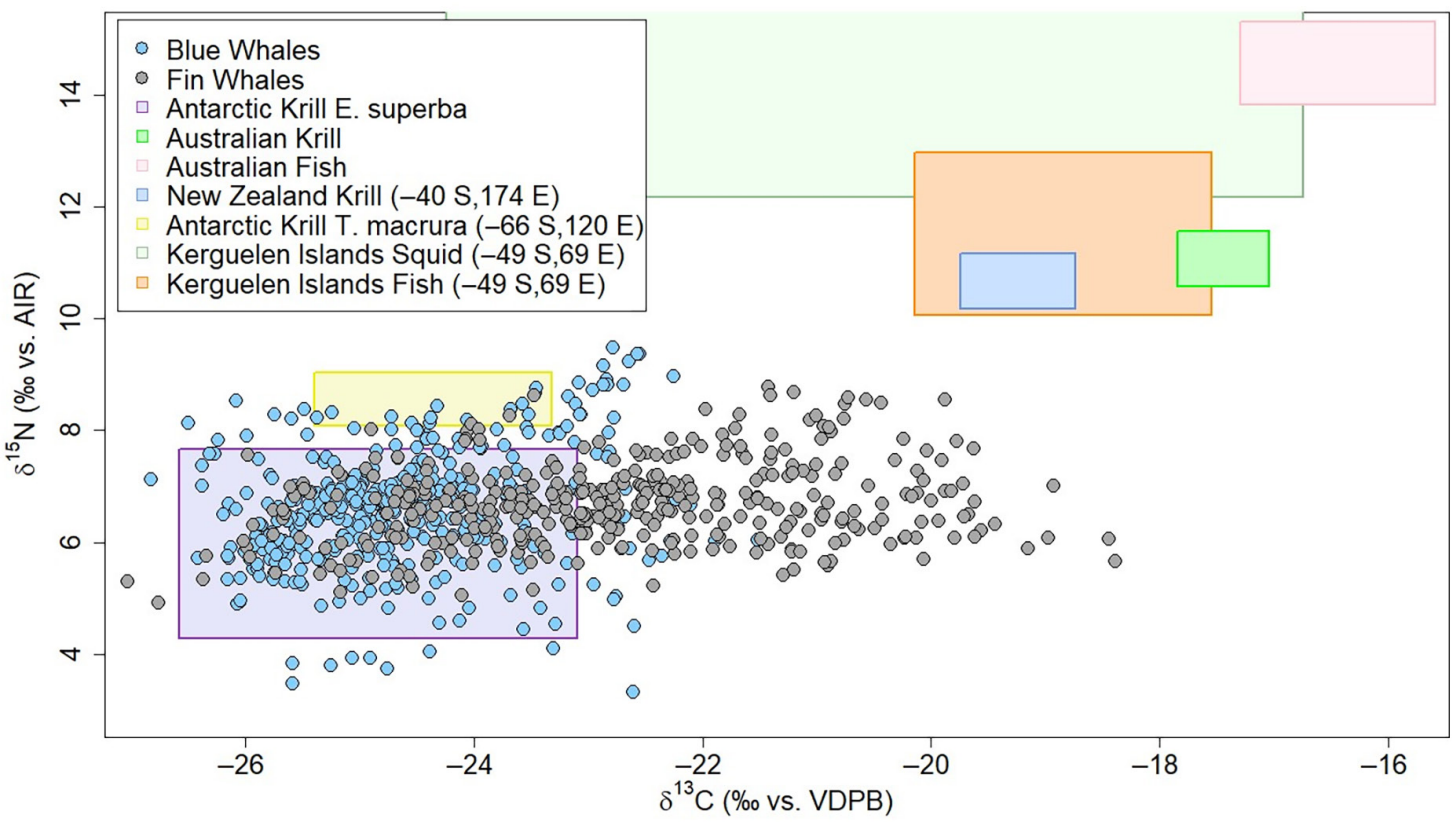
Plot of δ^13^C (x‐axis) and δ^15^N (y‐axis) values of baleen from all Antarctic blue (*Balaenoptera musculus intermedia*; blue circles) and fin whales (*Balaenoptera physalus*; gray circles). Stable isotope values for potential prey items are shown as highlighted squares (purple: Antarctic krill (*Euphausia superba*), green: Australian krill (multiple spp.), pink: Australian fish, blue: New Zealand krill (multiple spp.), yellow: Antarctic krill (*Thysanoessa macrura*), light green: Kerguelen Islands squid, and orange: Kerguelen Islands fish) based on values from Cherel et al. (2010), Guerreiro et al. (2015), Torres et al. (2015), Eisenmann et al. (2016), and Jia et al. (2016). Prey items have been corrected for fin whale baleen‐krill trophic fractionation (Borrell et al., 2012).

Using δ^13^C values from Espinasse et al. (2019) and the diet‐tissue fractionation calculations from Borrell et al. (2012) and Seyboth et al. (2018), 100% of blue whale δ^13^C values were within the range of values from the Southern Ocean (<−21.3‰; Figures 2, 3, and Figure A1), while 22.0% of fin whale δ^13^C values were within or near (seasonal variation: ±1.1‰) the range for the Subantarctic Front (>−20.2‰; Figures 2, 3 and Figure A2). When comparing all samples from the blue and fin whales to potential prey items, 81.0% of blue whale and 40.2% of fin whale samples had values within the range of Antarctic krill (*E. superba* and *T. macrura* combined) estimates (Figure 3). There were no samples within the range of the Australian, Kerguelen Islands, or New Zealand prey values (Figure 3). There were no significant differences in δ^13^C values between blue whales captured within or out of the Ross Sea (chi‐squared = 1.050, df = 1, *p* = .306).

**FIGURE A3:**
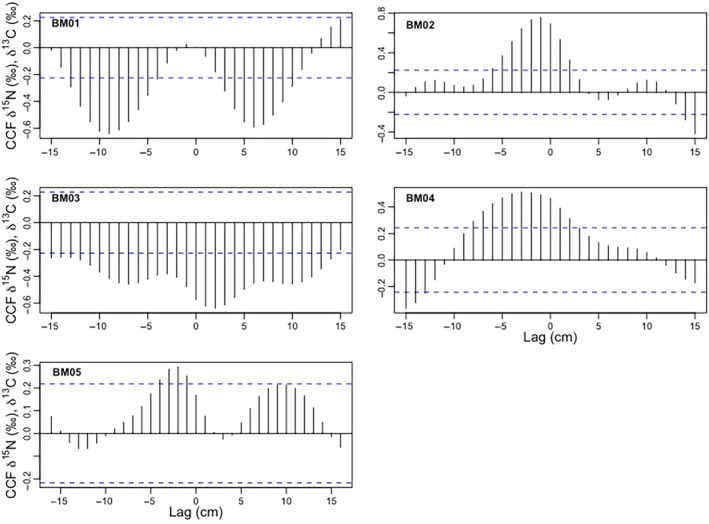
Cross‐correlation function graphs of δ^13^C and δ^15^N values for five Antarctic blue whales (*Balaenoptera musculus intermedia*; individuals BM01‐BM05). Stable isotope values are on the y‐axis, and the x‐axis shows the lag (sampling progression in centimeters along the plates). Values crossing the significance line (α = .05; dotted line) at lag 0 cm correspond to a significant cross‐correlation for the entire plate of an individual.

**FIGURE A4:**
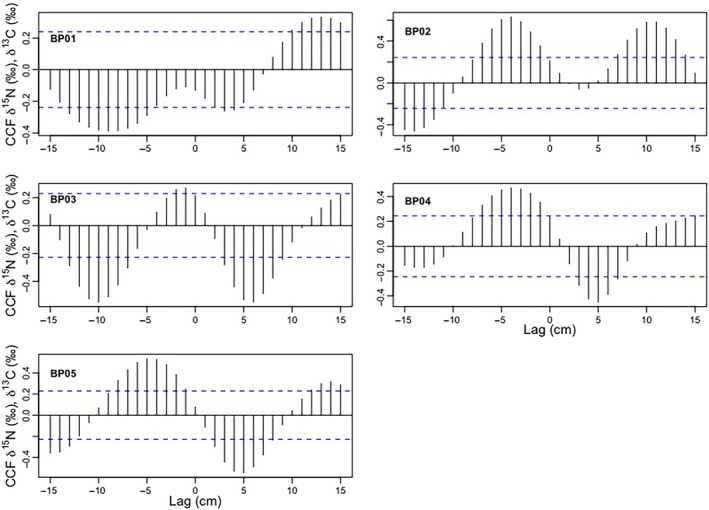
Cross‐correlation function graphs of δ^13^C and δ^15^N values for five fin whales (*Balaenoptera physalus*; individuals BP01‐BP05). Stable isotope values are on the y‐axis, and the x‐axis shows the lag (sampling progression in centimeters along the plates). Values crossing the significance line (α = .05; dotted line) at lag 0 cm correspond to a significant cross‐correlation for the entire plate of an individual.

There were two blue whales with significant positive correlations between δ^13^C and δ^15^N values, one with a significant inverse correlation, and two with no correlation (Figure A3). None of the fin whales showed any correlation between δ^13^C and δ^15^N values (Figure A4).

## 4 DISCUSSION

Collectively, the results of this study suggest that blue whales had different foraging behavior and migration patterns than fin whales in the Southern Ocean during the 1940s. This interpretation is based on physiological and isotopic metrics including baleen isotopic values and growth rates, stability and consistency of isotopic baleen cycles, and their inferred habitat use from isoscape data. The isotope signatures indicate that blue and fin whales overlap in their ecological niche more substantially during summer months (as inferred by isotope valleys) yet even then, show some niche differentiation. The five sampled blue whales appear to have spent more time within the Southern Ocean than the five sampled fin whales (Appendices C and D). Additionally, the fin whales seemed to be more regular in their migrations, fitting the classical model of longer seasonal movements to lower latitudes. Though our sample size of individuals is low (five adults from each species), a study design restriction necessitated by the large number of samples per individual, our study benefits from detailed repeated sampling from each individual (71–90 samples per individual), spanning multiple years and hence documenting multiple migration cycles. Our sample set also includes representatives of both sexes for each species, with females of varying reproductive states at death, and thus may offer some general insights into the foraging and migrations of 1940s Antarctic blue and fin whales in the relatively restricted area in which the study animals were killed.

### 4.1 Foraging patterns

#### 4.1.1 Foraging in the Southern Ocean

Under the assumption that the food webs of the Southern Ocean and surrounding areas have not substantially shifted in the last 70–80 years, the comparison of fin and blue whale isotope signatures with potential prey species (Figure 3) suggests that most blue whale samples and roughly half of fin whale samples were consistent with an Antarctic krill diet. The remaining fin whale samples largely correspond to migration or winter periods (discussed in the next section), but some summer season samples fall outside the values of Antarctic krill and may have been influenced by small contributions of other prey items (Figure 3). A recent study showed that an Atlantic population of fin whales switched to a more generalist diet when krill abundances were low, supporting the trophic flexibility of fin whales in other regions (Jory et al., 2021). It should also be noted that the potential prey species used in Figure 3 are from modern studies and have not been Suess corrected to the measured historic baleen plates. While the Suess Effect is negligible for the Southern Ocean, it likely would have an effect on the prey items from Australia, New Zealand, and the Kerguelen Islands (Eide et al., 2017). However, applying Suess correction to samples from these regions would elevate δ^13^C prey values shown in Figure 3, resulting in further isotopic separation between the baleen samples and those prey items outside of the Southern Ocean.

**FIGURE 4:**
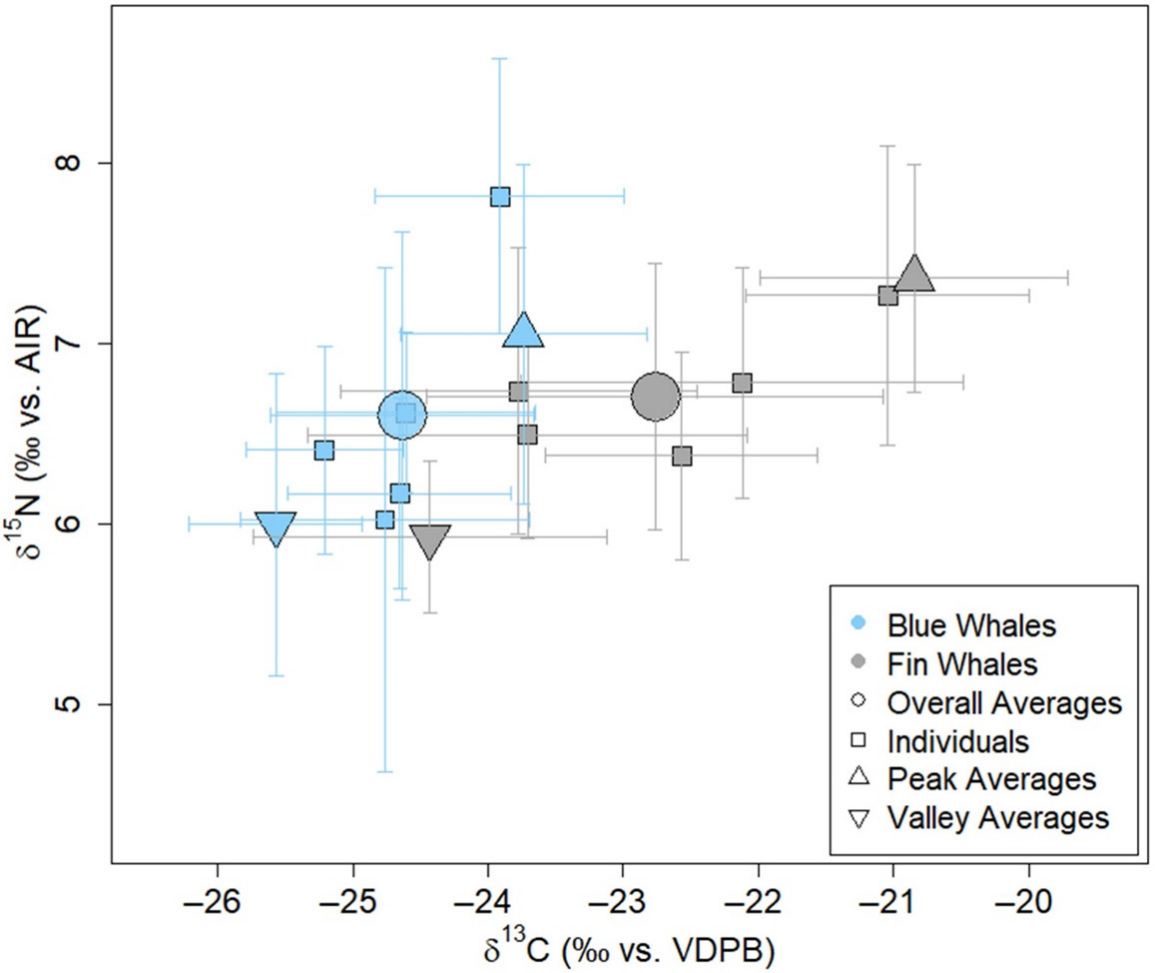
δ^13^C values and δ^15^N values showing five Antarctic blue whales (blue squares), five fin whales (gray squares), species overall averages (circles), peak (upright triangles), and valley averages (reverse triangles) with ±1 standard deviation bars.

Comparisons between the two species show that isotopic valleys (potential summer feeding ground signal) indicate a minimal difference in δ^15^N values (Figure 4). Because both species likely relied on a krill‐heavy diet while in the Southern Ocean (Figure 3; Laws, 1977), significant differences in prey composition are unlikely to have driven the isotopic carbon separation. The δ^13^C and δ^15^N values of both blue and fin whale valleys observed here fall within the range of *E. superba* (Figure 3), consistent with stomach content data showing dominance of *E. superba* and occasionally *T. macrura* (Jia et al., 2016; Nemoto, 1962; Yang et al., 2021). Fin whales have been observed to consume larger krill than blue whales (Laws, 1977; Santora et al., 2010), and larger adult Antarctic krill (*E. superba*) can have higher and more variable δ^15^N values than juveniles, yet with similar δ^13^C values (Polito et al., 2013). Thus, we might expect fin whales to show higher δ^15^N values than blue whales due to their consumption of larger krill and/or their more generalist diet, which can include higher trophic‐level schooling fish. However, the data presented here show similar blue and fin whale δ^15^N values, particularly while on summer feeding grounds (Figure 4). It is possible that the degree of isotopic difference between small and large‐bodied krill (δ^15^N: 1.5‰–2‰, Polito et al., 2013) may be too small to detect variations at the predator level, especially given the vast quantities and likely heterogeneity of krill that is consumed by these whales. Additionally, adult Antarctic krill may be carried north from the Weddell and Ross currents (Nemoto, 1962) and could still be consumed fin whales on migration towards the Subantarctic zone, maintaining the smaller difference in δ^15^N values between blue and fin whales.

Despite the similarity in δ^15^N values between blue and fin whale valleys, both the core (40%) and entire (95%) valley niche areas overlap only partially, suggesting some degree of resource partitioning while in the Southern Ocean. The difference in the niche areas is driven by a wider range of δ^13^C values for fin whales and may reflect a difference in foraging location. Fin whale baleen included elevated carbon values compared to blue whale samples, which could be associated with fin whales feeding farther north. Blue whales are more often observed near pack‐ice and are seldom observed foraging elsewhere (Laws, 1977; Nemoto, 1962), while fin whales have been observed farther from the ice edge often concentrating just south of the Polar Front around 55–62° S (Miyashita et al., 1995). The lower δ^13^C values of blue whales observed here could reflect either a higher latitude foraging location or regional difference. Blue whales were frequently observed and captured in the Ross Sea during these expeditions, a region with lower δ^13^C values than the waters around the Balleny Islands (Brault et al., 2018), while fin whales were not, potentially contributing further to the δ^13^C differences between species.

Overall, our data suggest that blue and fin whales had some degree of niche partitioning while in the Southern Ocean in the 1940s, when both species were at higher abundance than they are today. The much‐debated Krill Surplus Hypothesis suggests that the removal of large baleen whales from the marine ecosystem has resulted in a massive quantity of krill available to other, smaller predators (Laws, 1985). However, Savoca et al. (2021) estimated a much higher Antarctic krill consumption rate (430 million tonnes/year) by baleen whales and inferred higher krill abundance in the Southern Ocean prior to whaling than previously modeled (Barlow et al., 2008; Lavery et al., 2014). Regardless of the validity of the Krill Surplus Hypothesis and the krill production modeling, the isotope results described here indicate that geographical or habitat separation may have allowed these two species to minimize potential competition for krill and partition prey resources. It remains to be determined whether the two Antarctic species are performing similar niche partitioning today as reported in the Northern Hemisphere (García‐Vernet et al., 2021; Gavrilchuk et al., 2014).

#### 4.1.2 Foraging outside of the Southern Ocean

The traditional view of large baleen whale seasonal migrations from high‐latitude biologically productive feeding grounds to low‐latitude breeding and calving grounds has been challenged frequently for blue and fin whales (Busquets‐Vass et al., 2017; Geijer et al., 2016; Oleson et al., 2014). The isotopic peaks of Antarctic blue and fin whales diverged more in austral winter months (carbon peaks) than summer months (carbon valleys) (no overlap of core (40%) peak niche). While additional lines of evidence would be necessary to determine the reasons for these differences, fin whale peak values are consistent with foraging within the Subantarctic Front and possibly further north, while blue whale peak values are consistent with year‐round Southern Ocean locations (Borrell et al., 2012; Espinasse et al., 2019; Seyboth et al., 2018). Additionally, both the core (40%) and entire (95%) niches for blue whale peaks overlap with the core (40%) and entire (95%) niches for fin whale valleys, indicating that year‐round blue whale isotope values overlap with summer values of fin whales. If blue whales were to migrate to lower latitudes in the austral winter and continue to feed, it would be expected that their δ^13^C values would show an increase, a latitudinal pattern that has been previously documented in other marine predators (Eisenmann et al., 2016; Quillfeldt et al., 2005; Walters et al., 2014) as well as POM (Espinasse et al., 2019; Glew et al., 2021). Interestingly, the δ^15^N range of blue whales is wider than fin whales, a pattern driven by the peak (presumed winter) values from individuals Bm03 and Bm04. While individual differences in behavior may account for much of this wider range, it may also be impacted by seasonal variability in POM δ^15^N values or prey shifts in the winter months. A significant portion of fin whale samples fall outside the δ^13^C values that would be expected from a diet based on Antarctic krill (Figure 3) yet do not align with isotope data of other expected potential prey items. It is unlikely that seasonal changes within the Southern Ocean (i.e., if fin whales were to remain year‐round) explain all the variability in δ^13^C values between fin whale peaks and valleys. The seasonal changes of POM δ^13^C values within the Subantarctic Front and Antarctic have a range of about 1.5‰ (−22.41‰ to −21.18‰ and −28.19‰ to −26.65‰, respectively, yet potentially increase at the marginal ice zone; Espinasse et al., 2019). However, latitudinally there is a change of about 8‰ (−29.65‰ to −21.18‰) in POM from the Southern Ocean north to the Subtropical Convergence Zone. Therefore, the data presented here suggest that fin whales experience more seasonally varied isotopic input likely due to a greater degree of seasonal residence and foraging outside the Southern Ocean while blue whales show a higher degree of foraging specialization and dependence on Southern Ocean food webs year‐round (Mizue & Murata, 1951).

Isotopic patterns may also be influenced by physiological changes including temporary fasting behavior, yet this seems a less likely explanation for the observed δ^15^N oscillations. An increase in δ^15^N values may occur if an animal is fasting during migration, but this has been debated in mysticetes (Aguilar et al., 2014), which are well adapted to regular intervals of fasting and may not catabolize endogenous protein sources during periods of low food intake. Further, blue and fin whales often move between multiple biologically productive zones, making significant interannual adjustments in timing of arrival to optimize feeding success (Abrahms et al., 2019; Katsumata et al., 2023; Szesciorka et al., 2020) and may not experience sustained fasting periods (Burtenshaw et al., 2004; Lesage et al., 2017).

This historical evidence for foraging outside the Southern Ocean can be compared to contemporary observations. Antarctic blue and fin whales have been acoustically and visually detected in numerous highly productive areas in temperate regions outside of the Southern Ocean in recent decades including southern Australia (Aulich et al., 2019; Gill, 2002), Madagascar (Samaran et al., 2013), Chile (Herr et al., 2022; Sepulveda et al., 2018; Thomisch et al., 2016), and other locations throughout the southern Indian and Pacific Oceans as far north as 15° S (Balcazar et al., 2017) and evidence points to foraging behavior there. In contrast, as mentioned previously, acoustic detections of some blue whales have occurred year‐round in the Southern Ocean (Double et al., 2013; Širović et al., 2009) although virtually nothing is known about the prevalence of overwintering in this location, or potential foraging through winter months. Collectively, the isotopic, acoustic, and visual lines of evidence point towards a diversity of foraging strategies and locations for both blue and fin whales, including some blue whale individuals overwintering in the Southern Ocean both in past populations (WWII data presented here) and in more modern populations.

### 4.2 Migration

**FIGURE A5:**
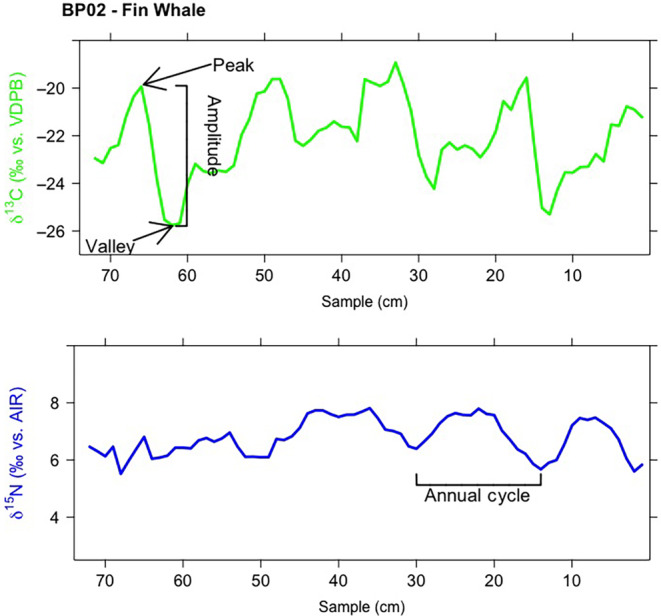
Schematic depicting the common terms associated with isotopic oscillations of a baleen plate, with data from fin whale Bp02 as an example. These include peak, valley, amplitude, and annual cycle. Peaks are the highest δ^13^C value of an oscillation, while valleys are the lowest. Amplitude is the difference between a δ^13^C peak and a valley. An annual cycle is the oscillation from one valley to the next based on δ^15^N values.

To assess migratory patterns, we used multiple metrics to examine changes in isotope values along the baleen plates. As carbon reflects sources of primary production and is useful for investigations of habitat use, carbon isotopes were considered more instructive for evaluating migratory behavior. Isotopic carbon amplitudes (Figure A5) were used to interpret the distance of migrations. Additionally, absolute values and patterns of carbon isotopes were evaluated in context of carbon isoscape data across the Southern Ocean (Espinasse et al., 2019).

#### 4.2.1 Blue whales

**FIGURE 5:**
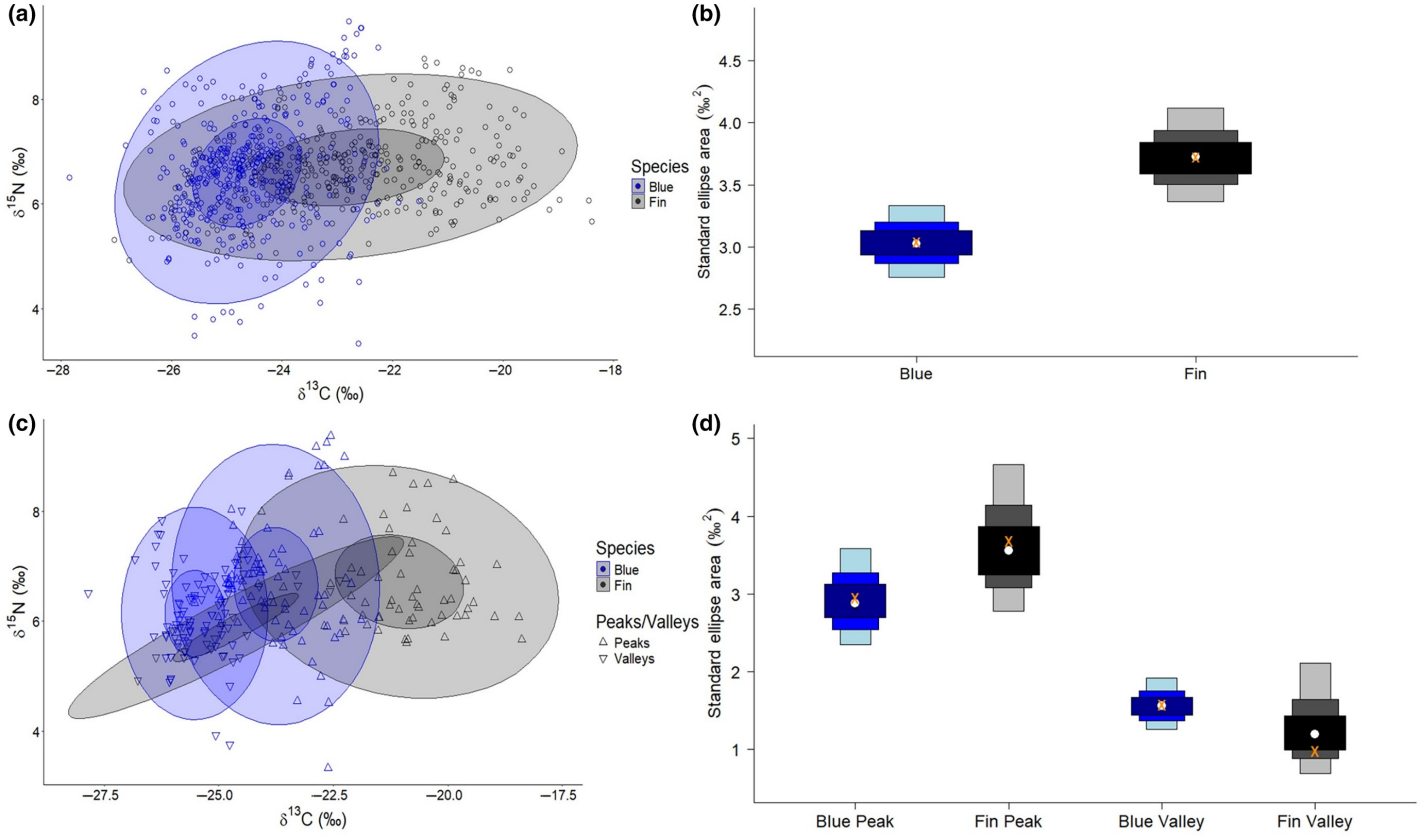
(a) SIBER plot showing bivariate stable isotope ratios of all blue (*Balaenoptera musculus intermedia*, blue) and fin whale (*Balaenoptera physalus*, gray) samples (dots). Bivariate ellipse areas estimated with 40% (smaller shaded ellipses) and 95% (larger shaded ellipses) of δ^13^C and δ^15^N values are shown (shading). (b) SIBER density plot of the standard ellipse area (‰^2^) for blue and fin whales as 50%, 95%, and 99% of the total data points (boxes). The SEAb values are shown as a white dot, and the SEAc values are shown as orange x's. (c) SIBER plot showing the standard ellipse areas and overlap of δ^13^C and δ^15^N values for peaks and valleys of blue (blue) and fin (gray) whales. (d) SIBER density plot of the standard ellipse area (‰^2^) for blue and fin whale peaks and valleys as 50%, 95%, and 99% of the total data points (boxes).

All blue whale δ^13^C values were within the range of values from the Southern Ocean (<−21.3‰; Figures 2, 3, and Figure A1). The average amplitude, or difference between peaks and valleys (Figure A5), for carbon isotopes along the baleen plates was smaller for blue whales (2.0‰) than fin whales (3.7‰). A smaller SIBER standard ellipse area (‰^2^) for blue whales (Table 2, Figure 5b) likely reflects a shorter migration, and/or movement between less isotopically distinct areas. Additionally, the overlap in isotopic space between blue whale peaks and valleys is substantially greater than the overlap between fin whale peaks and valleys (25% and 9%, respectively, of entire (95%) niche) demonstrating greater distinction between seasonal habitats for fin whales. One possible scenario is that blue whales make northward migrations, causing small variations in carbon peaks, yet remain within the Southern Ocean. This would be consistent with findings of some blue whale acoustic activity even in winter months around the Southern Ocean, even south of 69° S (Širović et al., 2009; Thomisch et al., 2016). Alternatively, multiple studies have provided support for longitudinal movements near the pack‐ice or islands (Kawamura, 1975; Mackintosh, 1965) with evidence of blue and fin whales moving between all sectors of the Southern Ocean (Attard et al., 2016; Brown, 1962; Sremba et al., 2012). Isoscape data from Espinasse et al. (2019) showed a change in δ^13^C values of 2.0‰ longitudinally across all the sectors, but no distinct gradient, making it challenging to assess longitudinal influence on the observed δ^13^C variation. The scale of longitudinal isoscape variability is consistent with the average amplitude of 2.0‰ found in these blue whale specimens, but the overall range of δ^13^C values for the blue whales in this study is slightly larger (2.9‰). This suggests that some of these whales, likely the two individuals with a range above 2.0‰, could have been migrating northward to the southern border of the Polar Frontal Zone and the Antarctic Zone (50–55° S, defined by Espinasse et al., 2019). The individuality and atypical nature of blue whale migration both within and out of the Southern Ocean has been documented by recent papers (Burtenshaw et al., 2004; Hucke‐Gaete et al., 2018; Lesage et al., 2017). In the northern hemisphere, multiple life history strategies and movement patterns have been observed, including residential non‐migratory populations, broad distributions throughout the year, or breeding and feeding in high latitudes through winter months (Blevins et al., 2022; Burtenshaw et al., 2004; Busquets‐Vass et al., 2017; Lesage et al., 2017). It is possible that combinations of latitudinal and longitudinal migrations are the cause of the less distinct annual cycles for the blue whales in this study (Table 1).

**TABLE 2:** SIBER data of Antarctic blue (*Balaenoptera musculus intermedia*) and fin (*Balaenoptera physalus*) whales.

	Blue overall	Fin overall	Blue peak	Fin peak	Blue valley	Fin valley
Sample size	421	381	5	5	5	5
SEA_b_	3.0	3.7	0.9	0.3	0.5	0.5
SEA_c_	3.0	3.7	1.5	0.3	0.7	0.7
Core niche overlap (40% contour)	9%		0%		12%	
Full niche overlap (95% contour)	42%		26%		24%	

*Note*: Modeled data include species' sample size, estimates of bivariate ellipses of isotope niche areas (SEA_b_ values and SEA_c_ values) of both species overall and compared by peaks and valleys of oscillations, and percentage of overlap of SEA_b_ isotopic niche areas using both the core (40%) and full (95%) niche.

#### 4.2.2 Fin whales

22.0% of fin whale δ^13^C values were within or near (seasonal variation: ±1.1‰) the range for the Subantarctic Front (>−20.2‰; Figures 2, 3 and Figure A2). Along with the more predictable annual cycles seen in the fin whale isotopic data, this suggests that they were making regular approaches to the Subantarctic Front (POM with diet‐tissue fractionation calculation: >−20.22‰; Borrell et al., 2012; Seyboth et al., 2018; Espinasse et al., 2019), and returning annually to the Southern Ocean, or North Antarctic Zone (POM with diet‐tissue fractionation calculation: <−21.32‰; Borrell et al., 2012; Seyboth et al., 2018; Espinasse et al., 2019). Furthermore, the wide range in δ^13^C values (range: −27.0‰ to −18.4‰) of these fin whales also supports a broader seasonal migration pattern. The isotopic evidence presented here is consistent with destinations near the Subantarctic Front or ~45° S (southern Australia or New Zealand), as suggested by some recent observations of modern populations (Aulich et al., 2019).

#### 4.2.3 Individual variation

Beyond species differences, substantial individual variation was apparent in multiple traits of the baleen plates. First, within species, there were differences in the number of isotope cycles found along the plates. It is possible that whales with only one or two measured cycles were not completing annual migrations. Because these specimens were sampled from biologically mature individuals (as estimated from body length; Lockyer, 1972; Branch, Abubaker, et al., 2007), it is not likely that these discrepancies are due to life stage, nor were there clear differences between males and females. Additionally, these cycles were longer than those of other individuals with regular annual cycles. This suggests that these individuals were spending more time either on feeding grounds, breeding grounds, or both (Figures A1 and A2).

Second, an examination of the individual patterns in δ^15^N shows differences between the species. For example, the amplitude of δ^15^N cycles within individual baleen plates was lower across the blue whale specimens compared to the fin whale specimens yet interindividual variability was slightly higher for blue whales (Figures A1 and A2). Contrastingly, individual fin whales all displayed a similar pattern to each other, resulting in a slightly lower range in overall δ^15^N for the species (Figure A2).

Third, there were differences in the relationship between the two isotopes across individuals. Mysticete whales with distinct summer and winter foraging areas, such as bowheads and North Atlantic right whales (*Eubalaena glacialis*), generally have consistent and well‐defined δ^13^C and δ^15^N cycles in baleen that are synchronized (Best & Schell, 1996; Lysiak et al., 2018; Matthews & Ferguson, 2015). In contrast, stable isotope studies using baleen of modern North Pacific blue whales have shown inconsistent patterns of annual cycles, possibly reflecting less prey switching across seasons or variation in migration behaviors (Blevins et al., 2022; Busquets‐Vass et al., 2017). In the present study, no fin whales showed any isotopic correlations between δ^15^N and δ^13^C, but individual blue whales exhibited both positive and negative isotopic synchronicity between carbon and nitrogen cycles, or none at all (Figures A3 and A4). Such diversity within the small number of blue whales sampled suggests that there may be high individual variability in patterns of migration and feeding. Conversely, in the fin whales sampled, the isotope cycles are generally offset, with carbon cycles lagging behind the nitrogen cycles for most of these fin whales (Figure A2). Unfortunately, there are very few studies on rorqual whales which focus on the synchronicity of δ^13^C and δ^15^N values (Hunt et al., 2018). This knowledge gap makes it difficult to interpret these patterns or compare our results to other populations.

## 5 CONCLUSIONS

The baleen plates analyzed in this study were collected when both species were still relatively abundant and decades before both were driven to commercial extinction by whaling in the Southern Ocean (Branch et al., 2004; Rocha et al., 2014) and before major ecosystem changes occurred due to climate change (Pinkerton et al., 2021; Rogers et al., 2020). The findings described here from whales harvested 70 years ago, while limited to a small number of individuals, suggest that Antarctic blue whale migrations in the 1940s were irregular and did not always extend to temperate ecosystems, while fin whale migrations were more consistent and likely more latitudinally extensive. Additionally, these species appeared to demonstrate some degree of resource partitioning, even within the Southern Ocean. Similar findings of niche differentiation amongst sympatric rorqual species have been documented previously both in and out of the Southern Ocean (Buss et al., 2022; Gavrilchuk et al., 2014; Ryan et al., 2014). Continued research on historic museum archives of historic baleen as well as modern baleen (e.g., from strandings) offers the potential to study trophic ecology and movement patterns in past populations as well as in present ones, providing opportunities to fill data gaps of “missing baselines,” as well as to evaluate potential changes occurring today. While such studies may initially involve a low n of individuals due to the time and effort required for analyses of dozens of samples from each individual, the resulting multi‐year datasets reveal a wealth of information about the predictability and regularity of individual feeding and movement. We encourage the study of more individuals, as well as addition of other concurrent analytical methods that can be applied to baleen, such as compound‐specific stable isotope analysis, endocrine analyses, and toxicological analyses. Ultimately, such studies can be combined into a growing dataset that may help reveal changes in foraging and migration patterns in the past and the present, as the Southern Ocean and other marine ecosystems continue to change.

## AUTHOR CONTRIBUTIONS

**Malia E. K. Smith:** Data curation (equal); formal analysis (equal); writing – original draft (equal); writing – review and editing (equal). **John J. Ososky:** Data curation (equal); resources (equal); writing – review and editing (equal). **Kathleen E. Hunt:** Conceptualization (supporting); writing – review and editing (equal). **William R. Cioffi:** Data curation (equal); writing – review and editing (equal). **Andy J. Read:** Supervision (equal); writing – review and editing (equal). **Ari S. Friedlaender:** Conceptualization (supporting); writing – review and editing (equal). **Matt McCarthy:** Conceptualization (supporting); writing – review and editing (equal). **Alyson H. Fleming:** Conceptualization (lead); data curation (equal); funding acquisition (equal); methodology (equal); supervision (lead); writing – original draft (equal); writing – review and editing (equal).

## CONFLICT OF INTEREST STATEMENT

The authors have no conflicts of interest to declare. We certify that the submission is original work and is not under review at any other publication.

## Data Availability

The data that support the findings of this study are openly available in Dryad at https://doi.org/10.5061/dryad.905qfttrt.

## APPENDIX A

### A.1

Specimen Details:

The USNM collection includes valuable metadata from the whaling logbooks, including the body length of harvested whales, catch location, sex, details of reproductive organs (size, weight, etc.), fetus lengths, stomach contents, blubber thickness, and the occurrence of parasites.

All individuals were captured in the southwest Pacific sector of the Southern Ocean south of New Zealand during the 1947/48 whaling season (Figure 1). For each species, two males, two pregnant females, and one non‐pregnant female were sampled (Table 1). Based on length measurements from the catch data, all individuals were presumed to be adults (Branch, Stafford, et al., 2007; Lockyer, 1972). All fin whales were between 20.7 and 21.6 m in length and blue whales were 22.5–25.8 m in length. The pregnant females selected had comparable fetus length measurements within species.

Analytical Method Details:

Samples were analyzed using a Thermo Scientific DELTA V Plus stable isotope mass spectrometer interfaced with Thermo Flash HT Plus EA/TCEA. Both U.S. Geological Survey (USGS) 40 (L‐Glutamic Acid; δ^13^C: −26.389‰, δ^15^N: −4.52‰) and 41a standards (L‐Glutamic Acid; δ^13^C: 36.56‰, δ^15^N: 47.6‰) were used at the beginning and end of every run and after every six samples.
